# Low CD4 count plus coma predicts cryptococcal meningitis in Tanzania

**DOI:** 10.1186/1471-2334-7-39

**Published:** 2007-05-10

**Authors:** Peter R Kisenge, Alexander T Hawkins, Venance P Maro, John PD Mchele, Ndealilia S Swai, Andreas Mueller, Eric R Houpt

**Affiliations:** 1 Department of Medicine, Kilimanjaro Christian Medical Centre, Moshi, Tanzania; 2 Division of Infectious Diseases and International Health, University of Virginia, Charlottesville, VA, USA; 3 Biotechnology Laboratory, Kilimanjaro Christian Medical Centre, Moshi, Tanzania; 4 Medical Mission Hospital, Department of Tropical Medicine, Hermann-Schell Str. 7, D-97074 Würzburg, Germany

## Abstract

**Background:**

Largely due to the lack of diagnostic reagents, the prevalence and clinical presentation of cryptococcal meningitis in Tanzania is poorly understood. This in turn is limiting the impact of increased fluconazole availability.

**Methods:**

We evaluated a cohort of 149 consecutive HIV-infected adult inpatients presenting with headache or altered mental status for clinical features, CD4 count, cryptococcal infection, and outcome. Cryptococcal meningitis was diagnosed via India ink and latex agglutination assay of CSF (*n *= 24 and 40 positive, respectively). Associations between cryptococcal meningitis and clinical features were evaluated by t-test. The sensitivity, specificity, and positive likelihood ratio of such features were determined.

**Results:**

Cryptococcal meningitis was associated with confusion, social withdrawal, seizures, fever, tachycardia, meningismus, oral candidiasis, and low Glasgow coma scales and CD4 count. CD4 count < 100/μl provided the highest sensitivity for the diagnosis (93%), coma (Glasgow coma scale ≤ 8) provided the highest specificity (84%), and the combination provided the highest positive likelihood ratio (3.8). All cryptococcal meningitis patients were initiated on 800 milligrams of fluconazole daily and 50% survived to discharge, however no clinical or laboratory findings correlated with prognosis.

**Conclusion:**

Cryptococcal meningitis is common among Tanzanian HIV inpatients presenting with headache or altered mental status. Purely clinical features are insensitive for establishing the diagnosis or prognosis. We advocate expanding laboratory capacity for cryptococcal antigen testing to maximize survival.

## Background

*Cryptococcus neoformans *is a leading AIDS-associated opportunistic infection and a major cause of adult meningitis in studies from Central African Republic, South Africa, Zambia, Zimbabwe [1-4]. In one report cryptococcal meningitis (CM) was the first AIDS-defining illness in 88% of patients [5]. The scope of the problem is less clear in Tanzania, where the literature is limited to one retrospective study that found *Cryptococcus *in 7% of (175/1144) CSF specimens [6]. A major reason for this lack of clarity is the lack of diagnostic reagents. For instance the Medical Stores Department, the main distributor of medical reagents to Tanzania, does not procure either India ink or cryptococcal antigen tests [7]. This leaves detection in the unlikely hands of expensive private imports. The need for accurate CM diagnosis is heightened by the increased availability of therapeutics to many parts of Africa, particularly fluconazole [8]. We therefore chose to examine a large cohort of Tanzanian HIV inpatients with central nervous system presentations to determine the local rate of CM using CSF cryptococcal antigen testing as the gold standard. We also sought to determine whether particular clinical features could predict the diagnosis as a stopgap measure until diagnostic reagents for CM become more widely available.

## Results

### Prevalence and clinical features of cryptococcal meningitis in Kilimanjaro region

This study took place on the inpatient medical ward of Kilimanjaro Christian Medical Centre, Moshi, Tanzania, a tertiary referral hospital. One hundred forty nine consecutive inpatients met enrollment criteria of headache (*n *= 86), altered mental status (Glasgow coma scale < 14; *n *= 5), or both (*n *= 58). Cryptococcal meningitis was diagnosed in CSF in 24 (17%) patients by India ink stain and in 40 (29%) by latex agglutination (*P *= 0.03 vs. India ink; sensitivity of India ink vs. latex agglutination 60%, specificity 100%). Several presenting features were statistically associated with the diagnosis of CM, including confusion, social withdrawal, seizures, fever, tachycardia, meningismus, oral candidiasis, and low Glasgow coma scales and CD4 count (Table 1). Spinal fluid indices were largely undiscerning. CM patients had a higher average pleocytosis (Table 1), however 50% of CM patients had an absence or paucity of CSF cells (a poor prognostic sign [2,12]). Additionally, 62% of the non-CM group had no CSF cells, indicating that meningitis was uncommon in many such patients with HIV and headache and/or altered mental status.

**Table 1:** Historical, examination, and laboratory findings in patients with cryptococcal meningitis.

	Cryptococcal meningitis (*n *= 40)	Other (*n *= 109)	*P*
Age (median, range)	41 (14–65)	37 (13–64)	NS
Female sex	45% (18)	45% (49)	NS
History			
Subjective fever	83% (33)	62% (68)	NS
Subjective wt loss	65% (26)	53% (58)	NS
Confusion	73% (29)	45% (49)	0.003
Vomiting	48% (19)	31% (34)	NS
Social withdrawal	43%(17)	18% (20)	0.004
Seizures	35% (14)	19% (21)	0.05
On antiretroviral therapy	18% (7)	24% (26)	NS
Physical examination			
Febrile (T >38.0°C)	73% (29)	47% (51)	0.006
Hypertensive (>140/90 mm Hg)	18% (7)	14% (15)	NS
Tachycardia	60% (24)	39% (42)	0.03
Meningismus	43% (18)	23% (28)	0.02
Oral thrush	48% (19)	24% (26)	0.008
Temporal wasting	50% (20)	35% (38)	NS
Abnormal GCS (≤ 14)	73% (29)	29% (32)	<0.0001
Coma (GCS ≤ 8)	50% (20)	16% (17)	<0.0001
Median GCS	8.5	15	<0.0001
Laboratory			
Leukocytes × 10^3^/μl (median)	6.4 ± 7.1 (5.4)	6.1 ± 5.6 (6)*	NS
Lymphocytes × 10^3^/μl (median)	2.0 ± 1.3 (1.7)	1.9 ± 1.2 (2.0)	NS
CD4 count/μl (median)	49.5 ± 45.2 (39)	190.0 ± 216.4 (89)	<0.0001
CD4 < 100/μl	93% (37)	51% (56)	<0.0001
Haemoglobin g/dl (median)	23.2 ± 29.1 (11.1)	17.9 ± 20.6 (12)*	NS
Platelets × 10^3^/μl (median)	161 ± 88 (141)	179 ± 104 (163)	NS
ESR (median)	87 ± 40 (93)	74 ± 39 (75)	NS
CSF†			
Pleocytosis % > 2/μl (*n*)	50% (20)	38% (41)	NS
Leukocytes (#/μl)†	63 ± 90	14 ± 23	0.03
Lymphocytes‡ (%)	53 ± 40	37 ± 34	NS
Neutrophils‡ (%)	47 ± 40	60 ± 34	NS
CSF protein (g/L)†	1.2 ± 2.3	0.7 ± 0.7	NS
CSF glucose (mmol/L)†	2.4 ± 1.3	3.0 ± 1.2	NS
CSF opening pressure (mmHg)	14.2 ± 10.6	9.8 ± 8.6	0.02
Outcome			
Survival to discharge	50% (20)	80% (87)	0.0008

Data shown are mean ± SD unless otherwise specified.

* no haemoglobin or leukocyte data from 1 other patient

† CSF cell counts, protein, glucose, and opening pressure results compare CM and only those other patients with meningitis, defined as CSF pleocytosis > 2/ml.

‡ CSF differential data available only from 12 CM and 11 non-CM patients.

### Sensitivity and specificity of clinical features for the diagnosis

**Figure 1:**
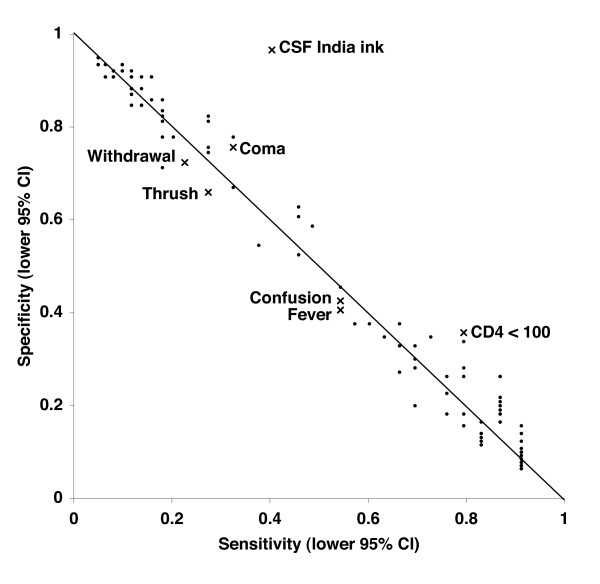
**Sensitivity and specificity of clinical features for the diagnosis of Cryptococcal meningitis**. The 95% confidence intervals of sensitivity and specificity for the clinical features associated with CM from Table 1 were plotted. Sensitivity and specificity for all possible combinations of clinical features were plotted as well. CSF India ink is shown for comparison. Diagonal line represents a Youden's J index of 0, such that tests to the left of the line are worthless (Youden's J ≤ 0) and tests to the far right are more worthwhile.

Although all patients received lumbar puncture and CSF cryptococcal antigen testing in this study, such tests are not always possible or available in Tanzania, and therefore we sought to evaluate whether clinical features alone could strengthen the likelihood of CM. Each clinical variable from Table 1 was examined for sensitivity and specificity alone and in combination (Figure 1). The most discriminating variables from Table 1 (social withdrawal, history of confusion, objective fever, oral candidiasis, coma, CD4 < 100/μl) gave the highest J scores (i.e., sensitivity + specificity - 1; data not shown) and are labeled in Figure 1. Notably, CD4 count < 100/μl provided the highest sensitivity (0.93, 95% CI 0.79 – 0.98; specificity 0.49) while coma provided the highest specificity (0.84, 95% CI 0.76 – 0.90; sensitivity 0.37). Of any variable or combination, CD4 count < 100/μl plus coma provided the highest J score, positive predictive value (0.58), and positive likelihood ratio (3.8, 95% CI 1.9 – 7.3). As expected, combining multiple clinical features, such as "confusion or fever or oral candidiasis" or "confusion and fever and oral candidiasis" could further drive sensitivity or specificity, respectively, but not both.

### Outcome

At the time of this study fluconazole was freely available, such that the physicians placed all patients with a positive CSF cryptococcal antigen test on 800 milligrams fluconazole per day orally or per nasogastric tube. Antiretroviral therapy (generally lamivudine, stavudine, and nevirapine) was either initiated or continued. Fifty percent (*n *= 20) of CM patients recovered and survived to discharge. CM was associated with mortality independent of CD4 count (18/37 CM patients with CD4 < 100/μl died vs. 15/56 non-CM patients with CD4 < 100/μl, *P *< 0.05). India ink positivity was not associated with a worse outcome (10/24 India ink positive patients died vs. 10/16 India ink negative CM patients; *P *= NS). Indeed, there was no statistical association between CM mortality and any of the clinical or laboratory indices measured (data not shown), only trends towards higher rates of hypertension (6/20 vs. 1/19; *P *= 0.09), higher opening pressure (median 14.5 vs. 10; *P *= 0.10), and higher erythrocyte sedimentation rates (median 106 vs. 78; *P *= 0.06) in the CM patients that died.

## Discussion

Our study shows that CM is common in northern Tanzania, occurring in 27% of HIV patients presenting with headache or AMS. The high rate is not surprising, although the magnitude of the problem had not been clear locally. Most reports on the severity of CM in Africa have originated from the South [1-3,5], leading some to question whether the disease is less common in the East and West [13,14]. Our results suggest the burden is also enormous in this region, similar to that seen in the Zimbabwe study [2].

The survival of our cohort was 50% to discharge. While less than the >80% survival seen in developed countries with the same 800 mg fluconazole regimen [15], our patients had far advanced disease and coma rates, and 50% survival is better than many reports from Africa [1,16,17]. Improved therapeutic regimens for CM are clearly needed, and one wonders if any added benefit would come from an additional agent such as flucytosine as seen in Uganda [18].

However we would advocate that improvements in therapeutics must be wed to improvements in diagnostics availability. During the time of study fluconazole was free (made available thanks to the Pfizer Diflucan Partnership Program), but this availability would have been aimless without cryptococcal antigen detection. Unfortunately, India ink exhibited a disappointing 60% sensitivity, worse than a clinical determination based on low CD4 count plus coma, therefore we are left advocating for increased availability of cryptococcal antigen tests (including use on serum [19]). As mentioned, the cryptococcal antigen test is presently not available through local dispensaries or Tanzania's Medical Stores Department, the country's main supplier of essential medicines and reagents. For the study tests were imported at a cost of $5.18 per test, admittedly not practical for a country whose per capita health expenditure is $12 [20]. However even at this high price per test, assuming fluconazole costs the present $0.97 per 100 mg in Tanzania (and will not always be donated), the diagnostic vs. therapeutic cost per CM survivor remains miniscule.

The finding that low CD4 count plus coma was a reasonably sensitive and specific feature for CM is unfortunate. First, clearly it requires CD4 quantitation, which although is presently more available in our region of Tanzania, is also technically demanding and expensive. Secondly, the finding of coma may provide high specificity but will inherently miss earlier and better-prognosis presentations [21]. Therefore while intuitively appealing to devise clinical management algorithms in settings with limited laboratory resources, we by no means advocate clinical diagnosis for this entity and would simply emphasize that the knowledge of CD4 < 100/μl plus coma in Tanzania should prompt strong consideration of CM.

Given CM's high mortality in Africa it seems prudent to examine preventive approaches instead of waiting until patients have advanced disease. We would predict that fluconazole prophylaxis in Tanzanian patients with CD4 < 100/μl, although costly, would prevent mortality (in contrast to the American study [22]). Alternatively, periodic screening of such patients with serum cryptococcal antigen could be comparator approach, accepting that this switches cost and infrastructure demands to the laboratory instead of the pharmacy.

There were a few final observations from our study. First, all patients underwent lumbar puncture, and this showed that many HIV patients with headache or altered mental status do not have meningitis. In many resource-limited settings where lumbar puncture is not performed due to erratic availability of equipment, empiric antibiotics are the norm, and our data would suggest this is often unnecessary and a waste of resources. Additionally, of the 149 admissions we document, 21% (24/113) of patients with CD4 < 200/μl were already on antiretroviral therapy, a starting point towards WHO and Global Fund goals.

## Conclusion

Cryptococcal meningitis is common among Tanzanian HIV inpatients presenting with headache or altered mental status. Given the insensitivity of purely clinical features for establishing the diagnosis or prognosis, we advocate expanding laboratory capacity for cryptococcal antigen testing to maximize survival.

## Methods

### Patients

Patients aged 13 – 65 were enrolled from the inpatient medical ward of the Kilimanjaro Christian Medical Centre (KCMC) from June 2005 – May 2006. Eligible patients included 162 consecutive HIV-positive inpatients with headache and/or altered mental status (Glasgow coma scale < 14). Thirteen patients were excluded from evaluation by lumbar puncture for concerns of papilloedema (3), cranial nerve palsies (2), or focal motor deficits (8). Informed consent for lumbar puncture was obtained from all participants, the University of Virginia Human Investigation Committee and the Kilimanjaro Christian Medical Centre Ethics Committee reviewed and approved the project, and all research was in compliance with the Helsinki Declaration. Patient histories came from the patient when possible else the patient's friends and/or family. All therapies were ordered by the consultant physician. Chart review demonstrated that all CM patients were initiated on 800 mg fluconazole (Diflucan, Pfizer, New York, NY) orally or per nasogastric tube for 2 weeks, followed by 400 mg orally for 4 weeks, followed by 200 mg orally thereafter. Of note, amphotericin B and flucytosine were not available in Tanzania at the time of this study, nor are they presently.

### Laboratory

Cryptococcal infection was assayed in cerebrospinal fluid by India ink testing (Becton Dickinson, Sparks, MD) and cryptococcal antigen latex agglutination (Murex Cryptococcus Test, Remel, Lenexa, KS). CD4 count was measured using the Coulter Manual CD4 Count kit (Beckman Coulter, Hialeah, FL). Haematology assays on CSF were performed at the KCMC Clinical laboratory with a Coulter counter and by microscopy.

### Statistics and diagnostic performance

Means and medians were compared using t test and Mann-Whitney test, respectively. All *P *values are two-tailed. Data shown are mean ± SD unless otherwise indicated. In accordance with STARD guidelines, the CSF cryptococcal antigen assay was utilized as the gold-standard test for the diagnosis of CM. Confidence intervals for proportions (sensitivity and specificity) were calculated using the Wilson score method [9]. Confidence intervals for positive and negative likelihood ratios were calculated as previously described [10]. The Youden's J index is used to rate the overall performance of a test and is calculated by (sensitivity + specificity - 1), where 0 is worthless and 1 is perfect. The positive likelihood ratio (= true positive rate/false positive rate) has been shown to be a superior method to convey the diagnostic value of a positive test result to clinicians [11].

## Abbreviations

CM = cryptococcal meningitis

KCMC = Kilimanjaro Christian Medical Centre

## Authors' contributions

PK conceived of the study and acquired the data. AH acquired the data. VM supervised the study. JM and NS carried out the CD4 counts and immunoassays. AM and EH assisted PK in the design of the study. EH provided funding and participated in data analysis and writing of the manuscript. All authors read and approved the final manuscript.

## Pre-publication history

The pre-publication history for this paper can be accessed here:


